# Concurrent bloodstream infection with *Lodderomyces elongisporus* and *Candida parapsilosis*

**DOI:** 10.1016/j.mmcr.2020.03.007

**Published:** 2020-04-01

**Authors:** Bryant Koh, Catriona Halliday, Raymond Chan

**Affiliations:** a Department of Infectious Diseases and Microbiology, New South Wales Health Pathology, Royal Prince Alfred Hospital, Missenden Street, Camperdown, 2050, Australia; b Centre for Infectious Diseases and Microbiology Laboratory Services, ICPMR, New South Wales Health Pathology, Westmead Hospital, Hawkesbury Road, Westmead, 2145, Australia

**Keywords:** Lodderomyces, Candida parapsilosis, Mixed candidaemia

## Abstract

We report the case of a 54-year-old patient with central venous catheter related mixed candidaemia with *Lodderomyces elongisporus* and *Candida parapsilosis*, who responded to line removal and anidulafungin therapy.

Mixed candidaemia was detected on Candida chromogenic agar. Identification of the two isolates was confirmed by MALDI-TOF MS (Bruker). Antifungal susceptibility testing revealed different antifungal MICs. This is the first reported case of mixed *Lodderomyces* candidaemia and outlines laboratory methodology to aid diagnosis and management.

## 1 Introduction

*Lodderomyces elongisporus* is an uncommon cause of candidaemia, usually associated with immunosuppression or intravenous access devices [[1], [2], [3], [4], [5], [6]]. It is one of four species recognised within the *Candida parapsilosis* complex; other species include *C. parapsilosis*, *C. orthopsilosis* and *C. metapsilosis* [[7], [8], [9]]. These four species are physiologically similar and identification by biochemical methods is unreliable, with both Vitek 2 (bioMérieux) and API 20C (bioMérieux) systems generally mis-identifying isolates as *C. parapsilosis* [1]. *L. elongisporus* is currently on neither database. On chromogenic *Candida* agar, *L. elongisporus* colonies are typically turquoise while other species within the *C. parapsilosis* complex are pink/lavender [[1], [2], [3],^6^]. MALDI-TOF mass spectrometry or PCR amplification and DNA sequencing of the internal transcribed spacer region and/or D1/D2 domain of the rRNA gene accurately identify all four species within the *C. parapsilosis* complex [10].

Mixed candidaemia is uncommon, with rates of 3%–5% [[11], [12], [13]]. Correct identification of mixed infection and speciation of organisms isolated ensures appropriate management. We report the case of a patient with line-associated mixed candidaemia with *Lodderomyces elongisporus* and *Candida parapsilosis*, who responded to anidulafungin and line removal.

## 2 Case

A 54-year-old woman was admitted to hospital with recurrent stoma malfunction and prolapse requiring surgical revision, on a background of prior total colectomy and ileostomy formation for chronic pseudo-obstruction, and short gut syndrome. A long-term Hickman line for total parenteral nutrition (TPN) was last replaced 10 months prior to admission, with a history of previous Hickman line infections secondary to enteric and skin flora.

**Fig. 1:**
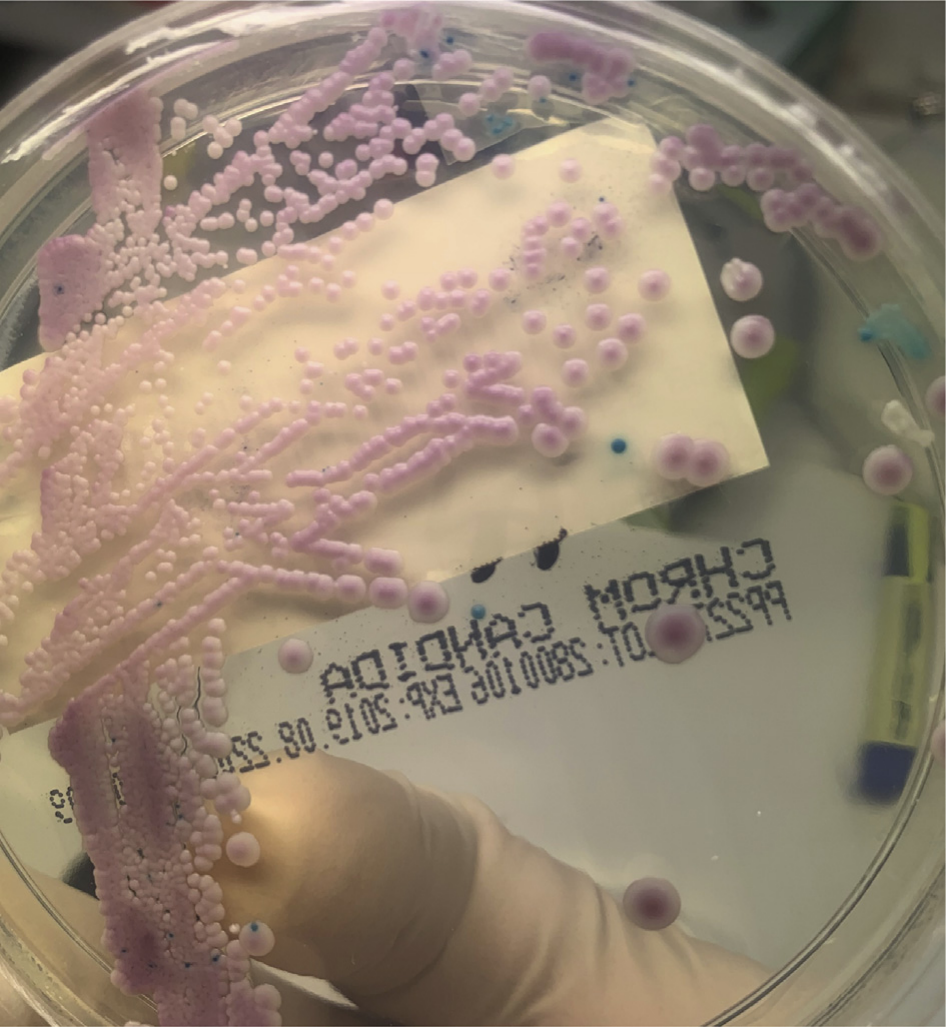
Chromogenic *Candida* Agar (ThermoFisher Scientific) plate with pink (*C. parapsilosis* complex) and turquoise (*L. elongisporus*) colonies. (For interpretation of the references to colour in this figure legend, the reader is referred to the Web version of this article.)

The patient had a fever on day +1 of admission and a set of blood cultures was collected from the patient's Hickman line. Budding yeasts were identified on microscopy of the anaerobic BacT/ALERT® (bioMérieux) blood culture bottle after 23 hours incubation. Empiric anidulafungin (200mg IV loading dose, followed by 100mg IV daily) was commenced. The subsequent isolate was identified on MALDI-TOF (Bruker) as *C. parapsilosis* (score = 2.1) after sub-culture. Yeasts were also identified in the aerobic bottle after 28.5 hours incubation. After 2 days incubation of sub-culture of the aerobic bottle, 2 morphologic colony types were noted on chocolate agar, subsequently identified on MALDI-TOF as *C. parapsilosis* (score 2.26) and *L. elongisporus* (score 2.04). *L. elongisporus* colonies were observed to have lighter pigmentation than *C. parapsilosis*. Sub-culture of the aerobic blood culture bottle revealed pink and turquoise colonies after overnight incubation on Chromogenic *Candida* Agar (ThermoFisher Scientific) (Fig. 1). MALDI-TOF of the green/blue colonies identified these as *L. elongisporus* (score 2.15). Antifungal susceptibility testing of both isolates was performed using the Sensititre™ YeastOne™ YO10 plate (ThermoFisher Scientific) (Table 1).

**Table 1:** Antifungal susceptibility testing results.

	*C. parapsilosis* MIC (μg/mL)	*L. elongisporus* MIC (μg/mL)
Amphoterecin B	0.25	0.12
Anidulafungin	0.5	0.015
Caspofungin	0.25	0.008
Fluconazole	0.5	0.12
Flucytosine	<0.06	0.06
Itraconazole	0.03	0.03
Micafungin	1	0.015
Posaconazole	0.03	0.015
Voriconazole	0.008	0.008

*C. parapsilosis* was also identified in the aerobic bottle of a second blood culture collected on day +2 of admission after 24 hours incubation. The Hickman line was removed on day +6 of admission, and culture of the tip grew >100 colony-forming units of *C. parapsilosis* (identified by MALDI-TOF) after 24 hours incubation. A PICC was inserted for ongoing TPN.

Anidulafungin was continued for 14 days following Hickman line removal (ceased on day +20). There was no growth in blood cultures collected day +10 and subsequently, and the patient had clinical improvement with resolution of fever following Hickman removal.

## 3 Discussion

Mixed candidaemia is difficult to identify using non-differential media alone and may have important impact when selecting antifungal therapy. When yeast is observed in positive blood cultures by microscopy, our laboratory routinely sub-cultures onto Chromogenic *Candida* agar to aid in identification of mixed candidaemia. Although two phenotypes were observed on chocolate agar, identification of mixed yeasts was more readily seen on chromogenic agar.

Antifungal susceptibility patterns of the two isolates were similar but not identical, with low minimum inhibitory concentrations (MICs) to all agents tested. This is consistent with the few published isolates [[1], [2], [3], [4], [5], [6],^9^]. Although categorically comparable, MICs for anidulafungin and micafungin had more than fourfold difference between our isolates. Local Australian Therapeutic Guidelines [14] and the Infectious Diseases Society of America guidelines [15] recommend empirical echinocandins as first-line therapy for candidaemia. Fluconazole may be used instead in select non-critically ill patients unlikely to have a fluconazole-resistant *Candida* species. As the patient tolerated anidulafungin and had a clinical response, the decision was made not to alter therapy once antifungal susceptibility results were available.

In addition, consideration of removal of central venous catheters if present must be part of treatment of candidaemia, particularly in non-neutropaenic patients or those in whom a line is considered the source of infection [16]. Our laboratory uses the method described by Maki et al. for processing of central venous catheter tips [17]. *L. elongisporus* was not identified on tip culture of the Hickman line in our case, which may have been due to the relatively low inoculum of *L. elongisporus* compared with *C. parapsilosis* or misidentification as catheter tip specimens are not routinely plated onto chromogenic agar. As the Hickman line was the likely source of candidaemia, removal was an important part of therapy.

## Ethical form

This research did not receive and specific grant from funding agencies in the public, commercial, or not-for-profit sectors. The authors have no conflicts of interest to disclose. Consent was obtained from the patient to publish this case report.

## Declaration of competing interest

There are none.

## References

[1] Lockhart S.R., Messer S.A., Pfaller M.A., Diekema D.J. (2008). *Lodderomyces elongisporus* masquerading as *Candida parapsilosis*as a cause of bloodstream infections. J. Clin. Microbiol..

[2] Daveson K.L., Woods M.L. (2012). *Lodderomyces elongisporus* endocarditis in an intravenous drug user: a new entity in fungal endocarditis. J. Med. Microbiol..

[3] Ahmad S., Khan Z.U., Johny M., Ashour N.M., Al-Tourah W.H., Joseph L. (2013). Isolation of *Lodderomyces elongisporus* from the catheter tip of a fungemia patient in the Middle East. Case Rep Med.

[4] Hantanaka S., Nakamura I., Fukushima S., Ohkusu K., Matsumoto T. (2016). Catheter-related bloodstream infection due to *Lodderomyces elongisporus*. Jpn. J. Infect. Dis..

[5] Lee H.Y., Kim S.J., Kim D., Jang J., Sung H., Kim M.N. (2018). Catheter-related bloodstream infection due to *Lodderomyces elongisporus* in a patient with lung cancer. Ann Lab Med.

[6] Al-Obaid K., Ahmad S., Joseph L., Khan Z. (2018). *Lodderomyces elongisporus*: a bloodstream pathogen of greater clinical significance. New Microbe and New Infect.

[7] James S.A., Collins M.D., Roberts I.N. (1994). The genetic relationship of *Lodderomyces elongisporus* to other ascomycete yeast species as revealed by small-subunit rRNA gene sequences. Lett. Appl. Microbiol..

[8] Tavanti A., Davidson A.D., Gow N.A.R., Maiden M.C.J., Odds F.C. (2005). *Candida orthopsilosis* and *Candida metapsilosis spp. nov.* to replace *Candida parapsilosis* Groups II and III. J. Clin. Microbiol..

[9] Tay S.T., Na S.L., Chong J. (2009). Molecular differentiation and antifungal susceptibilities of *Candida parapsilosis* isolated from patients with bloodstream infections. J. Med. Microbiol..

[10] Asadzadeh M., Ahmad S., Hagen F., MEis J.F., Al-Sweih N., Khan Z. (2015). Simple, low-cost detection of *Candida parapsilosis* complex isolates and molecular fingerprinting of *Candida* orthopsilosis strains in Kuwait by ITS region sequencing and amplified fragment length polymorphism analysis. PloS One.

[11] Jensen J., Munoz P., Guinea J., Rodriguez-Creixems M., Pelaez T., Bouza E. (2007). Mixed fungemia: incidence, risk factors, and mortality in a general hospital. Clin. Infect. Dis..

[12] Al-Rawahi G.N., Roscoe D.L. (2013). Ten-year review of candidemia in a Canadian tertiary care centre: predominance of non-*albicans Candida* species. Can. J. Infect Dis. Med. Microbiol..

[13] Pulimood S., Ganesan L., Alangaden G., Chandrasekar P. (2002). Polymicrobial candidemia. Diagn. Microbiol. Infect. Dis..

[14] (2019 Jun). Candida species sepsis (candidaemia) [published 2019 Jun]. eTG Complete [digital].

[15] Pappas P.G., Kauffman C.A., Andes D.R., Clancy C.J., Marr K.A., Ostrosky-Zeichner L. (2016). Clinical practice guideline for the management of candidiasis: 2016 update by the Infectious Diseases Society of America. Clin. Infect. Dis..

[16] Andes D.R., Safdar N., Baddley J.W., Playford G., Reboli A.C., Rex J.H. (2012). Impact of treatment strategy on outcomes in patients with candidemia and other forms of invasive candidiasis: a patient-level quantitative review of randomized trials. Clin. Infect. Dis..

[17] Maki D.G., Weise C.E., Sarafin H.W. (1997). A semiquantitative culture method for identifying intravenous-catheter-related infection. NEJM.

